# Propensity of Tampons and Barrier Contraceptives to Amplify *Staphylococcus aureus*Toxic Shock Syndrome Toxin-I

**DOI:** 10.1155/S1064744994000542

**Published:** 1994

**Authors:** Philip M. Tierno, Bruce A. Hanna

**Affiliations:** Departments of Microbiology and Pathology New York University School of Medicine New York University Medical Center Bellevue 4W1, 560 First Ave. New York NY 10016 USA

## Abstract

*Objective:* Although the incidence of reported cases of toxic shock syndrome (TSS) has declined in recent years, the disease continues to occur in menstruating women using the newer, less-absorbent tampons or barrier contraceptives. Extant tampons and other vaginal devices were tested for the ability to induce TSS toxin-1 (TSST-1) by a TSS strain of *Staphylococcus aureus* MN8, a known high-toxin producer. Tested for the first time were 20 varieties of tampons, including 2 all-cotton brands newly introduced in the United States, a polyurethane contraceptive sponge, a latex diaphragm, and a polymer menstrual collection cup.

*Methods:* All products were washed in sterile distilled water prior to use to reduce the effect of leachable chemicals. Duplicate experiments with unwashed products were also performed. Entire tampons and other test products were immersed in brain heart infusion broth plus yeast extract (BHIY) and inoculated with *S. aureus* MN8, a known TSST-1 producer. After incubation, the culture supernatants were assayed for TSST-1 by gel immunodiffusion.

*Results:* Except for all-cotton tampons, greater amounts of TSST-1 were detected in the supernatant fluid of washed tampons than detected in those which were not washed. While TSST-1 levels in unwashed non-cotton tampons ranged from 0.5 to 8 μg/ml, when these products were washed, TSST-1 levels increased to 2–32 μg/ml. In all-cotton tampons, whether washed or not, there was no detectable TSST-1.

*Conclusions:* The propensity for all-cotton tampons not to amplify TSST-1 in vitro suggests they would lower the risk for tampon-associated TSS.


Infectious Diseases in Obstetrics and Gynecology 2:140–145 (1994)
© 1994 Wiley-Liss, Inc.

### Propensity of Tampons and Barrier Contraceptives to Amplify *Staphylococcus aureus* Toxic Shock Syndrome Toxin-1

**Philip M. Tierno, Jr., and Bruce A. Hanna**

*Departments of Microbiology and Pathology, New York University School of Medicine, New York University Medical Center, New York, NY*

Abstract

***Objective:*** Although the incidence of reported cases of toxic shock syndrome (TSS) has declined in recent years, the disease continues to occur in menstruating women using the newer, less-absorbent tampons or barrier contraceptives. Extant tampons and other vaginal devices were tested for the ability to induce TSS toxin-1 (TSST-1) by a TSS strain of *Staphylococcus aureus* MN8, a known high-toxin producer. Tested for the first time were 20 varieties of tampons, including 2 all-cotton brands newly introduced in the United States, a polyurethane contraceptive sponge, a latex diaphragm, and a polymer menstrual collection cup.

***Methods:*** All products were washed in sterile distilled water prior to use to reduce the effect of leachable chemicals. Duplicate experiments with unwashed products were also performed. Entire tampons and other test products were immersed in brain heart infusion broth plus yeast extract (BHIY) and inoculated with *S. aureus* MN8, a known TSST-1 producer. After incubation, the culture supernatants were assayed for TSST-1 by gel immunodiffusion.

***Results:*** Except for all-cotton tampons, greater amounts of TSST-1 were detected in the supernatant fluid of washed tampons than detected in those which were not washed. While TSST-1 levels in unwashed non-cotton tampons ranged from 0.5 to 8 μg/ml, when these products were washed, TSST-1 levels increased to 2–32 μg/ml. In all-cotton tampons, whether washed or not, there was no detectable TSST-1.

***Conclusions:*** The propensity for all-cotton tampons not to amplify TSST-1 in vitro suggests they would lower the risk for tampon-associated TSS. © 1994 Wiley-Liss, Inc.

Key words

TSS, toxins, menstrual

The incidence of reported cases of menstrual toxic shock syndrome (TSS) that fulfill the strict epidemiologic case definition criteria has declined since high-absorbency fibers such as polyacrylate rayon, polyester, and carboxymethylcellulose were removed from tampons.[1] Nonetheless, the disease continues to occur, especially in young menstruating women using the newer, less-absorbent tampons made of viscose rayon with and without cotton. Women using barrier contraceptives are also at increased risk for TSS. Whole tampons or their components have been reported to amplify the production of TSS toxin-1 (TSST-1) by TSS strains of *Staphylococcus aureus* grown in their presence.[2–8] Until recently, however, all-cotton tampons were not available in the United States for either analysis or use. A comparison of the propensity for these newer tampon products, including the newly avail-

Address correspondence/reprint requests to Dr. Bruce A. Hanna, Bellevue 4W1, NYU Medical Center, 560 First Ave., New York, NY 10016.

Received May 13, 1994
Accepted July 21, 1994

Basic Science Article able all-cotton tampons as well as other vaginal devices, to amplify TSST-1 production is the subject of this report.

### MATERIALS AND METHODS

One hundred sixty-four extant tampons and 1 extinct control tampon representing 20 varieties, 6 Today contraceptive sponges, 6 Tassaway menstrual collection cups, and 4 Ortho All-Flex diaphragms were assayed for the ability to induce TSST-1 by *S. aureus* MN8, a known high-toxin producer. Prior to the assay, all products were soaked overnight in 100 ml of sterile distilled water at room temperature in order to reduce the leachable chemicals contained within the products. After washing, whole tampons and sponges were aseptically removed, squeezed, then washed again in sterile distilled water for 1–2 h. Whole products were squeezed again, then immersed into 40 ml of brain heart infusion broth with 1% yeast extract (BHIY) which contained approximately $10^5$ cfu (colony forming units)/ml of *S. aureus* MN8, a known high-toxin producer originally recovered from a case of TSS. Since menstrual cups and diaphragms are made of non-absorbent material, they were not squeezed after washings. A duplicate set of experiments with unwashed products was also performed as were experiments with BHIY controls. All experiments were incubated overnight in an atmosphere of 5% $CO_2$ at 37°C without shaking, then assayed for TSST-1 by a gel immunodiffusion procedure previously described.[2] Final bacterial populations were determined by a standard viable plate count. The Mann-Whitney test was used to compare all sets of 3 or more data points with still controls; the null hypothesis was rejected with $P < 0.01$. The anticipated conclusion is that tampons (except cotton varieties), diaphragms, and sponges affect toxin production.

### RESULTS

The composition and relative absorbency of the vaginal devices tested are listed in Table 1. The results of experiments to detect TSST-1 in the supernatant of *S. aureus* cultivated in the presence of washed and unwashed products as well as controls are listed in Table 2. The recorded ranges of TSST-1 are an average for each product style and type. These data indicate that the level of toxin production was dependent upon the product tested. In general, except for all-cotton tampons, greater amounts of TSST-1 were detected in the supernatant fluid of washed tampons than detected in those that were not washed. While TSST-1 levels in unwashed tampons ranged from 0.5 to 8 μg/ml, when these products were washed, TSST-1 levels increased to 2–32 μg/ml. The greatest stimulation of TSST-1, by extant brand tampon types, was observed with OB tampons (9.3–18.7 μg/ml), Kotex (6.7–13.3 μg/ml), Playtex (6.7–9.3 μg/ml), Tampax (4–8.0 μg/ml), and CO-OP (3.3–6.7 μg/ml). The 2 all-cotton tampon brands, Terra Femme and Natracare, produced no measurable toxin whether washed or unwashed. A comparison of all sets of 3 or more data points with the Mann-Whitney test showed all of the non-cotton tampons tested affected TSST-1 production compared with both the still broth control ($P < 0.01$) and the all-cotton tampons ($P < 0.01$). To ensure that the lack of TSST-1 was not strain specific, an equal number of Terra Femme all-cotton tampons were tested using a second strain of TSST-1 producing *S. aureus* (No. 3984). As with the MN8 strain, TSST-1 was not detected in any of the experiments.

The Today contraceptive sponge, when washed, produced 1–4 μg/ml of TSST-1, while the unwashed product with nonoxynol-9 produced 0–0.5 μg/ml of toxin. The nonoxynol-9 is inhibitory to *S. aureus* as evidenced by the 2-log decrease in the viable bacterial counts of unwashed sponges compared with the washed product as well as with other products tested. This finding is in agreement with our previously reported observations.[2,3] The polyester and carboxymethylcellulose tampon (Rely) yielded 20–80 μg/ml of TSST-1 when unwashed but only 5–10 μg/ml after washing. This is less than previously reported. Since this tampon was manufactured almost 15 years ago, it has likely undergone deterioration as was apparent during the experiments in which the washed product showed signs of polyester disintegration, discoloration, crumpling, and loss of sponginess. Further, as is characteristic of the carboxymethylcellulose, the Rely tampon could not be squeezed free of fluid and remained in a gelled state, i.e., did not display negative absorption.

The Tassaway vaginal menstrual cups are made from a non-absorbent elastomeric polymer. *S. aureus* MN8 produced no TSST-1 when grown in the presence of Tassaway, whether washed or un-

TABLE 1. Chemical composition of products tested[a]

| Product | Composition: Pledget | Composition: Outer wrap | Composition: Cord | S-L-F | Absorbency (g) |
|---|---|---|---|---|---|
| Tampax | | | | | |
| Regular-Compak | Rayon | Rayon | Cotton | + | 6–9 |
| Super | Cotton/rayon | Rayon | Cotton | + | 9–12 |
| Super-Plus | Rayon | Rayon | Cotton | + | 12–15 |
| OB | | | | | |
| Regular | Rayon/cotton | PNS | Rayon/cotton | + | 6–9 |
| Super | Rayon/cotton | PNS | Rayon/cotton | + | 9–12 |
| Super-Plus | Rayon/cotton | PNS | Rayon/cotton | + | 12–15 |
| Playtex | | | | | |
| Reg/do/po | Rayon | None | Cotton | + | 6–9 |
| Reg/po | Rayon | None | Cotton | + | 6–9 |
| Regular-Ultimate | Rayon/cotton | Rayon | Cotton | + | 6–9 |
| Super-Ultimate | Rayon/cotton | Rayon | Cotton | + | 9–12 |
| Kotex | | | | | |
| Regular | Cotton/rayon | PNS | Rayon/polyester/cotton | + | 6–9 |
| Super | Cotton/rayon | PNS | Rayon/polyester/cotton | + | 9–12 |
| Super-Plus | Cotton/rayon | PNS | Rayon/polyester/cotton | + | 12–15 |
| Terra Femme | | | | | |
| Regular | Cotton | None | Cotton | – | 10 |
| Super | Cotton | None | Cotton | – | 13 |
| Natracare | | | | | |
| Regular | Cotton | None | Cotton | – | 10 |
| Super | Cotton | None | Cotton | – | 13 |
| CO-OP | | | | | |
| Regular | Rayon/cotton | None | Cotton | + | 10 |
| Super | Rayon/cotton | None | Cotton | + | 13 |
| Rely | | | | | |
| Super | Polyester/cmc | Polyester | Polyester | + | 19 |
| Today | Polyurethane | None | Nylon | + | 20 |
| Tassaway | Elastomeric polymer | None | None | – | 0 |
| Ortho All-Flex | Latex | None | None | – | 0 |

[a]S-L-F = surfactant-leachable-fragrance present; PNS = present not specified; Reg/do/po = regular deodorant portable; Reg/po = regular portable; cmc = carboxymethylcellulose.

washed. Ortho diaphragms, made from latex rubber, produced 0.5 μg/ml of TSST-1 when unwashed and 0.75 μg/ml when washed. An adherent film of staphylococcal growth was evident on the surface of all test diaphragms. TSST-1 was detected in the shaker control broth (3–4 μg/ml) but not in the still broth control. Shaker broth controls produced the highest viable staphylococcal counts and were at least 1 log higher than the counts of any tampon experiments. The viable bacterial counts of the washed tampons were slightly higher than the counts of the unwashed product.

### DISCUSSION

Previous reports have shown that vaginal tampons, especially those rich in synthetic fibers, provide the physical and chemical parameters that permit TSST-1 production to varying degrees dependent upon product composition.[2–8] Numerous physical, chemical, and ecologic factors are reported to affect TSST-1 production by strains of *S. aureus* grown in the presence of vaginal products.[2–6] Currently marketed tampons manufactured in the United States (Table 3) are made from rayon or rayon/cotton blends and have been shown to amplify TSST-1 in vitro. Since an all-cotton tampon was not previously available, comparisons were not possible until now. We have found that all-cotton tampons, whether washed or unwashed, did not produce detectable TSST-1 compared with the other tampons, which confirms previous results using tampon-sized pledgets of surgical cotton.[2,3] This difference in toxin production is likely because cotton provides fewer physical-chemical factors that favor TSST-1 production. Cotton is less absorbent, has less surface area, does not effectively concen-

TABLE 2. Effect of products on the production of TSST-1[a]

| | Unwashed | | | | Washed | | | |
|---|---|---|---|---|---|---|---|---|
| | | | TSST-1 (μg/ml) | | | | TSST-1 (μg/ml) | |
| Product | No. | cfu/10$^{-8}$ | Range | Average | No. | cfu/10$^{-8}$ | Range | Average |
| Tampax | | | | | | | | |
| Regular-Compak | 3 | 4.3 | 0.5–2 | 1.2 | 3 | 4.5 | 4 | 4.0 |
| Super | 2 | 5.8 | 1–4 | 2.5 | 2 | 6.1 | 4–8 | 6.0 |
| Super-Plus | 3 | 3.4 | 2–4 | 3.3 | 3 | 6.0 | 8 | 8.0 |
| OB | | | | | | | | |
| Regular | 3 | 4.7 | 2–4 | 3.2 | 3 | 4.4 | 4–16 | 9.3 |
| Super | 3 | 4.2 | 4 | 4.0 | 3 | 6.1 | 8–16 | 13.3 |
| Super-Plus | 3 | 5.4 | 4–8 | 6.7 | 3 | 7.1 | 8–32 | 18.7 |
| Playtex | | | | | | | | |
| Reg/do/po | 3 | 2.2 | 1–2 | 1.3 | 3 | 4.1 | 4–8 | 6.7 |
| Reg/po | 3 | 6.3 | 1–4 | 2.3 | 3 | 4.6 | 8 | 8 |
| Regular-Ultimate | 3 | 4.0 | 1–2 | 1.7 | 3 | 4.0 | 4–8 | 6.7 |
| Super-Ultimate | 3 | 3.9 | 2–4 | 3.3 | 3 | 4.8 | 4–16 | 9.3 |
| Kotex | | | | | | | | |
| Regular | 3 | 3.6 | 1–2 | 1.7 | 2 | 4.2 | 4–8 | 6.7 |
| Super | 3 | 4.4 | 2–4 | 3.3 | 3 | 4.8 | 8 | 8 |
| Super-Plus | 3 | 5.6 | 2–4 | 3.3 | 3 | 6.4 | 8–16 | 13.3 |
| Terra Femme | | | | | | | | |
| Regular | 20 | 3.1 | 0 | 0 | 3 | 4.1 | 0 | 0 |
| Super | 20 | 2.7 | 0 | 0 | 3 | 7.0 | 0 | 0 |
| Natracare | | | | | | | | |
| Regular | 5 | 2.9 | 0 | 0 | 3 | 5.2 | 0 | 0 |
| Super | 5 | 2.3 | 0 | 0 | 3 | 6.2 | 0 | 0 |
| CO-OP | | | | | | | | |
| Regular | 5 | 2.5 | 1–4 | 2 | 3 | 5.9 | 2–4 | 3.3 |
| Super | 5 | 3–4 | 1–2 | 1.4 | 3 | 6.6 | 4–8 | 6.7 |
| Rely Super | 8 | 4.2 | 20–80 | 35 | 2 | 6.9 | 5–10 | 7.5 |
| Today | 3 | 0.03 | 0–0.5 | <0.5 | 3 | 1.1 | 1–4 | 3.0 |
| Tassaway | 3 | 4.2 | 0 | 0 | 3 | 4.1 | 0 | 0 |
| Ortho All-Flex | 2 | 6.8 | 0.5 | 0.5 | 2 | 7.9 | 0.75 | 0.75 |
| Broth Controls | | | | | | | | |
| Shaker (200 rpm) | 15 | 65.0 | 2–4 | 3.0 | 6 | 68.3 | 2–8 | 4.0 |
| Still | 17 | 2.6 | 0 | 0 | 6 | 3.7 | 0 | 0 |

[a]Reg/do/po = regular deodorant portable; Reg/po = regular portable.

trate protein in aqueous solutions, and provides a less viscous environment compared with the synthetics previously removed from tampons (carboxymethylcellulose, polyester, polyacrylate rayon), even those which are still used in tampon manufacture (viscose rayon).[3] None of the all-cotton tampons tested contain surfactants or finishing agents, some of which are known to affect TSST-1 production.[2–4,9,10]

In the vaginal milieu, many factors may influence the polymicrobial environment and the products of bacterial metabolism. Independent of chemical composition, factors such as tampon structure, absorbency or fluid capacity, air content, and surface area may affect TSST-1 production.[3] Nonetheless, it has been reported that cotton actually

TABLE 3. Marketing/distributing country of vaginal products tested

| Product | Company | Country |
|---|---|---|
| Tampax | Tambrands, Inc. | United States |
| OB | Personal Products Co. | United States |
| Playtex | Playtex Family Products Corp. | United States |
| Kotex | Kimberly-Clark Corp. | United States |
| Terra Femme | Bio Business International Corp. | Canada |
| Natracare | Bodywise, Ltd. | England/Sweden |
| CO-OP | CWS | England |
| Rely[a] | Proctor-Gamble | United States |
| Today | Whitehall Laboratories | United States |
| Tassaway | Personal Care Products | United States |
| Ortho All-Flex | Ortho | United States |

[a]No longer on the market.

adsorbs TSST-1.[11] Cotton loses that adsorbing ability, however, when combined with rayon.[11] It is likely, therefore, that all-cotton tampons lower the risk for TSS since they do not amplify TSST-1 in vitro and have useful adsorbing affinity for that toxin. This is especially important since menstrual TSS associated with synthetic-fiber tampons continues to occur despite the decreased absorbency; the elimination of carboxymethylcellulose, polyacrylate rayon, and polyester; and the increased awareness of the role of tampons in TSS.[1]

The viable bacterial counts for washed tampons were higher than those of the unwashed products probably because inhibitory leachables were removed by the washing process. Likewise, TSST-1 increased after the tampons, except the all-cotton variety, were washed. When tampons are placed in an aqueous solution, they have been shown to leach out various inorganic and organic chemicals which increases the viscosity of that solution.[12] Washing the vaginal products prior to testing more accurately reflects their conditions of use. Since the vaginal mucosa is known to readily absorb chemicals,[13] such leachables as surfactants and other finishing agents used in products may likewise be absorbed, diluted, or inactivated as they mix with vaginal fluids.[2,3] Since some leachable chemicals may inhibit toxin production or the growth of *S. aureus*, that effect may be mollified by the washing, hence producing the slightly higher viable cell counts and the significant increase in TSST-1 levels. The unwashed product, therefore, does not reflect a product's true propensity for amplifying TSST-1 since interference from some leachable chemicals could be expressed within a closed-test system.

It is not surprising that no toxin was found with the Tassaway menstrual cups. They are made from an elastomeric polymer, a non-absorbent product that is apparently inert. In addition, they do not allow for adherence of *S. aureus* to their surface, as occurred with the latex diaphragm. Latex diaphragms, although non-absorbent, allow for adherence and, as such, can act as a nidus for *S. aureus* proliferation on both sides of the diaphragm. While the concave side facing the cervix may be protected by the antibacterial properties of the spermicide, the convex side facing the vagina has no such protection. Diaphragms have been reported to cause TSS,[14] which is attributed to the growth of *S. aureus* in the presence of semen[15] and prolonged retention of the diaphragm which may increase the staphylococcal count by more than 3 logs.[14] Nonetheless, TSS has been associated with the use of latex diaphragms[14,15,18] as well as other latex products.[16]

It is possible that, although the Today contraceptive sponge is impregnated with nonoxynol-9, a spermicide with known antimicrobial properties, this effect can be mollified when the sponge absorbs protein-rich semen or vaginal fluids. The effect of the spermicide will be offset if the product remains in place for an extended period of time, thereby removing some of the inhibiting agent. This is not unlike the bacteriologic growth conditions of a blood-laden tampon. It has been reported that TSST-1 production dramatically increases when the *S. aureus* is grown with the Today contraceptive sponge in a medium containing blood or semen.[17] This report also indicated that significant TSST-1 production occurs even under anaerobic conditions in the presence of the sponge. While these studies do not mimic in vivo conditions, they do indicate that TSST-1 strains of *S. aureus* should be able to grow and produce toxin in situ in the presence of blood, semen, or vaginal secretions, as has been evidenced by the TSS cases reported in the literature.[11] A comparative analysis of women who developed TSS while using either contraceptive sponges or diaphragms and controls indicates an increased risk of TSS.[18] The matched odds ratio for Today contraceptive sponge users was 10.5 and the odds ratio for the diaphragm users was 11.7.

Thus, it appears that women who are without protective levels of antibody to TSST-1 and use non-all-cotton tampons, diaphragms, or contraceptive sponges are at the greatest risk for TSS.[11] The remaining imperative is for epidemiologic surveillance to verify these findings with all-cotton tampons. Until that time, in light of the occurrence of TSS among women using non-cotton tampons and the current data strongly indicating that all-cotton fibers would reduce the risk of TSS, we would be prudent to suggest the exclusive use of all-cotton fibers in catamenial devices.

### REFERENCES

1. Centers for Disease Control: Reduced incidence of menstrual TSS—United States 1980–1990. MMWR 39: 421–423, 1990.

2. Tierno PM, Hanna BA: In vitro amplification of toxic shock syndrome toxin-1 by intravaginal devices. Contraception 31:185–194, 1985.
3. Tierno PM, Hanna BA: The ecology of toxic shock syndrome. Rev Infect Dis 2:5182–5186, 1989.
4. Lee AC, Cross BA, Bergdoll MS: Investigation by syringe method of effect of tampons on production in vitro of toxic shock syndrome toxin-1 by *Staphylococcus aureus*. J Clin Microbiol 25:87–90, 1987.
5. Robbins RN, Reiser RF, Hehl GL, Bergdoll MS: Production of TSST-1 by *Staphylococcus aureus* as determined by tampon disk-membrane agar method. J Clin Microbiol 25:1446–1449, 1987.
6. Reiser RF, Hinzman SJ, Bergdoll MS: Production of TSST-1 by *Staphylococcus aureus* restricted to endogenous air in tampons. J Clin Microbiol 25:1450–1452, 1987.
7. Mills JT, Parsonnet J, Tsai Y-C, Kendrick M, Hickman RK, Kass EH: Control of production of TSST-1 by the magnesium ion. J Infect Dis 151:158–161, 1985.
8. Holland KT, Ingham E, Gowband G: TSS: The effect of phase materials on the physiology of *Staphylococcus aureus*. Postgrad Med J 61:39–43, 1985.
9. Schleivert PM, Deringer JR, Kim MH, Projan SJ, Novick R: Effect of glycerol monolaurate on bacterial growth and toxin production. Antimicrob Agents Chemother 36: 626–631, 1992.
10. Wong-Lee AC: Factors affecting growth of *Staphylococcus aureus* and production of TSST-1. Ph.D. dissertation. University Microfilms Inc., Ann Arbor, MI 48106, 1987.
11. Bergdoll MS: Tampon involvement in menstrual TSS. In Bergdoll MA, Chesney PJ (eds): Toxic Shock Syndrome. Boca Raton, FL: CRC Press, pp 187–221, 1991.
12. Center for Medical Device Analysis: Measurement of Leachables From Tampons. Bethesda, MD: U.S. Food and Drug Administration, 1981.
13. Hartman C: The permeability of the vaginal mucosa. Ann NY Acad Sci 83:318–327, 1947.
14. Given WD: TSS associated with diaphragm use. W Va Med J 82:171–173, 1986.
15. Baehler EA, Dillon WP, Mryja DM, Neter E: The effects of prolonged retention of diaphragms on colonization of *S. aureus* of the lower genital tract. Fertil Steril 39:162–166, 1983.
16. Allen ST, Liland JB, Nichols CG, Glew RH: TSS associated with use of latex packing. Arch Intern Med 150: 2587–2588, 1990.
17. Faich GA, Sobel S, Bilstad J: TSS and the vaginal contraceptive sponge. JAMA 251:1015–1018, 1984.
18. Schwartz B, Gaventa S, and TSS Study Group: Nonmenstrual TSS associated with barrier contraceptives: Report of a case control study. Rev Infect Dis 11:S43–S47, 1989.
